# Variable fluid properties analysis for thermally laminated 3-dimensional magnetohydrodynamic non-Newtonian nanofluid over a stretching sheet

**DOI:** 10.1038/s41598-023-30233-7

**Published:** 2023-02-24

**Authors:** Noreen Sher Akbar, A. Al-Zubaidi, S. Saleem, Shami A. M. Alsallami

**Affiliations:** 1 DBS&H CEME, National University of Sciences and Technology, Islamabad, Pakistan; 2 Department of Mathematics, College of Science, King Khalid University, Abha, 61413 Saudi Arabia; 3 Department of Mathematical Sciences, College of Applied Science, Umm Al-Qura University, Makkah, 21955 Saudi Arabia

**Keywords:** Mathematics and computing, Nanoscience and technology

## Abstract

This article is mainly focused on the viscous flow of cu-water/Methanol suspended nanofluids towards a three-dimensional stretching sheet reformed by magnetohydrodynamic phenomenon. The viscous effect is considered as temperature dependent with water treated as a base fluid. Similarity conversions are employed to set forth the non-linear equations of this physical problem. An innovative model for 3D analysis for cu-water/Methanol nanofluid with an irregular viscosity is presented in the present study. Reynold’s model of viscosity is considered in the present study. Moreover, shooting technique is employed to elaborate the non-linear coupled governing equations with the relevant boundary conditions. The physical interpretation of these numerical calculations is presented through a graphical specimen of velocity, Nusselt number, temperature, and skin friction etc. The results of present model are showing quality harmony with the results of existing model. This model is being used for manipulating and designing the surfaces such as stretching/shrinking wrapping and panting devices in nanotechnology. The results also show the significant changes in flow characteristics with changing the value of stretching parameter. It is observed that with an increasing in nanoparticles volume fraction boundary layer thickness decreases. Further, it is also observed that with an increase in viscosity parameter, temperature increases because here we are considering temperature dependent viscosity.

## Introduction

There are many industrial requirements to manufacture the products which optimize the time, cost, space and pollution etc. to fulfill the worldwide needs. Works in the field of Nano science and nanotechnology has recently aided in the optimization of constraints in industrial and engineering requirements. Nowadays, there are many refinements and improvements in the field of nanoscience and technology which are reported by researchers and scientists. Nanofluid dynamics is a part of nanoscience where we study the effects of nanoparticles volume, energy distribution and accumulation with base fluid. The restoration of electronic equipment, vehicles temperature reduction, nuclear catalyst all includes the influence of nanotechnology. Generally, it reported that nanoparticles enhance the thermal conductivity of working fluids.

Das et al.^1^ had illuminated the dramatic thermal enhancement effects of nanofluids with practical applications in material sciences, chemical engineering and physical sciences as well. Wang and Arun^2^ had interpreted the impacts of nanofluids on the flow distribution and characteristics in both free and forced convection problems. Trisaksri and Wongwises^3^ had presented multiple techniques in their study to highlight the addition of nanoparticles and their impact on thermal enhancement. Murshed et al.^4^ had conducted a comparison of the experimental and analytical results of Nanofluid addition and thermal enhancement effects. Wang and Mujumdar^5^ had also narrated the theoretical aspects in addition to the numerical calculations for thermal enhancement and nanofluid addition. Kakac and Pramuanjaroenkij^6^ had conveyed a review study on the nanofluids heat distribution impacts on the forced convection problems. Wen et al.^7^ had provided practical applications on this fascinating topic of heat distribution enhancement with Nanofluids, as well as its limitations. Yu and Xie^8^ had explained the multiple methods of nanofluid preparation, the stability of nanofluids and some practical applications in their research review. Some other key research works that illustrate the impact of heat distribution enhancement via Nano fluids and their applications are^9–13^. Akbar et al.^14,15^ had provided a mathematical model of the nanofluid effects on a vertically placed stretching surface under a magnetic field and uneven viscous impacts. They had also highlighted the applications of CNTs in biotechnology and micro-electronic appliances. Sheikholeslami and Chamkha^16^ had numerically investigated the electric field effects on the natural convection of nanofluids via finite element technique. Uddin et al.^17^ had utilized the Lie-group conversions for the first time for a bio-convection flow problem with nanofluidic impacts. Sheikholeslami and Chamkha^18^ had studied the uneven MHD effects on the lid driven cavity flow problem with enhanced nanofluid heat transfer effects. Kothandapani and Prakash^19^ had modeled the nanofluid effects on the peristaltic flow with radiation and porosity impacts. Brownian motion and thermophoresis are two key points of their research work. The magnetic field impact, hydro-thermal treatment via nanofluids, applications of nanofluids in micro-fluidic appliances and, cilia effects are narrated in following studies^20–25^.

After a thorough review of the literature on nanofluids, it is observed that two-dimensional flow of nanofluids is reported in most of the studies, which do not cover the all industrial and nanotechnological applications. Some authors^26–31^ reported the three-dimensional flow analysis for nanofluids which are more relevant in application point of view in comparison to two-dimensional and one dimensional flows. Hayat et al.^26^ had investigated the impact of nanofluid heat distribution on Visco-elastic models; Mahanthesh et al.^27^ had reported 3D analysis for heat distribution impact of nanofluids with nonlinear radiations; Kolsi et al.^28^ discussed the entropy generation nanofluids in three dimensional Buoyancy-Induced Flow; Khan et al.^29^ presented numerical results for a heated deformable surface with nanofluids heat distribution; Khan et al.^30^ extended their work for Burgers nanofluid. Zhu et al.^31^ numerically analyzed the convective heat distribution impacts enhanced via nanofluid addition inside a wavy conduit containing aluminum oxide and ethylene glycol nano particles. They have obtained the numerical solutions and found that the thermal conductivity of silver-water nanofluid enhances with increasing nanoparticle volume fraction and temperature but decreases for larger sized nanoparticles.

The investigation of boundary-layer theory for a stretching plane has intrigued many researchers and industry people because of its widespread applications in industry and nanotechnology. Such models are applicable in production of paper, plastic sheets firms, and fiber, glass production etc. Motivated by the huge range of industrial applications on stretching sheets flow problems with nanofluids, some authors^32–34^ have presented the 3D flow analysis of nanofluids considering different stretching geometries viz. bidirectional stretching surface, slandering stretching and stretching sheet. The MHD impacts in a 3D flow analysis for nanofluids through a nonlinear stretching surface and slandering stretching sheet was modeled by Nadeem et al.^35^. Further literature can be view and analyzed through Refs.^36–39^. Venkata et al.^40^ had discussed the impact of Soret and Dufour on MHD Casson fluid flow past a stretching surface with convective diffusive conditions. They observed that depreciate temperature and appreciate concentration with the upsurge in Sorret number.

Numerous theoretical, experimental and numerical investigations on nanofluids have been reported in the last few years to progress the new technologies for the increasing heat distribution production of base fluids. There are many sorts of nanoparticles that are distributed in base fluid and enhanced thermal conductivity is attained. Methanol is one of the most applicable nanoparticles that result in a very effective way to boost the thermal conductivity. Inspired from the combined effect of magnetic field and Methanol nanoparticles with Reynolds model of viscosity, a three-dimensional flow analysis for the stretching sheet is presented in this analysis. The motivation behind this work is that there is no previous work which reported combined analysis of nanoparticles with Reynolds mode of viscosity. As it is temperature dependent viscosity model. This model is being utilized in many industrial applications. Like sanitary fluid transport, blood pumps in heart lungs machine and transport of corrosive fluids etc. As for fluids in industry and in engineering prospectus fluids have variable fluid properties.

## Formulation of the problem

**Figure 1:**
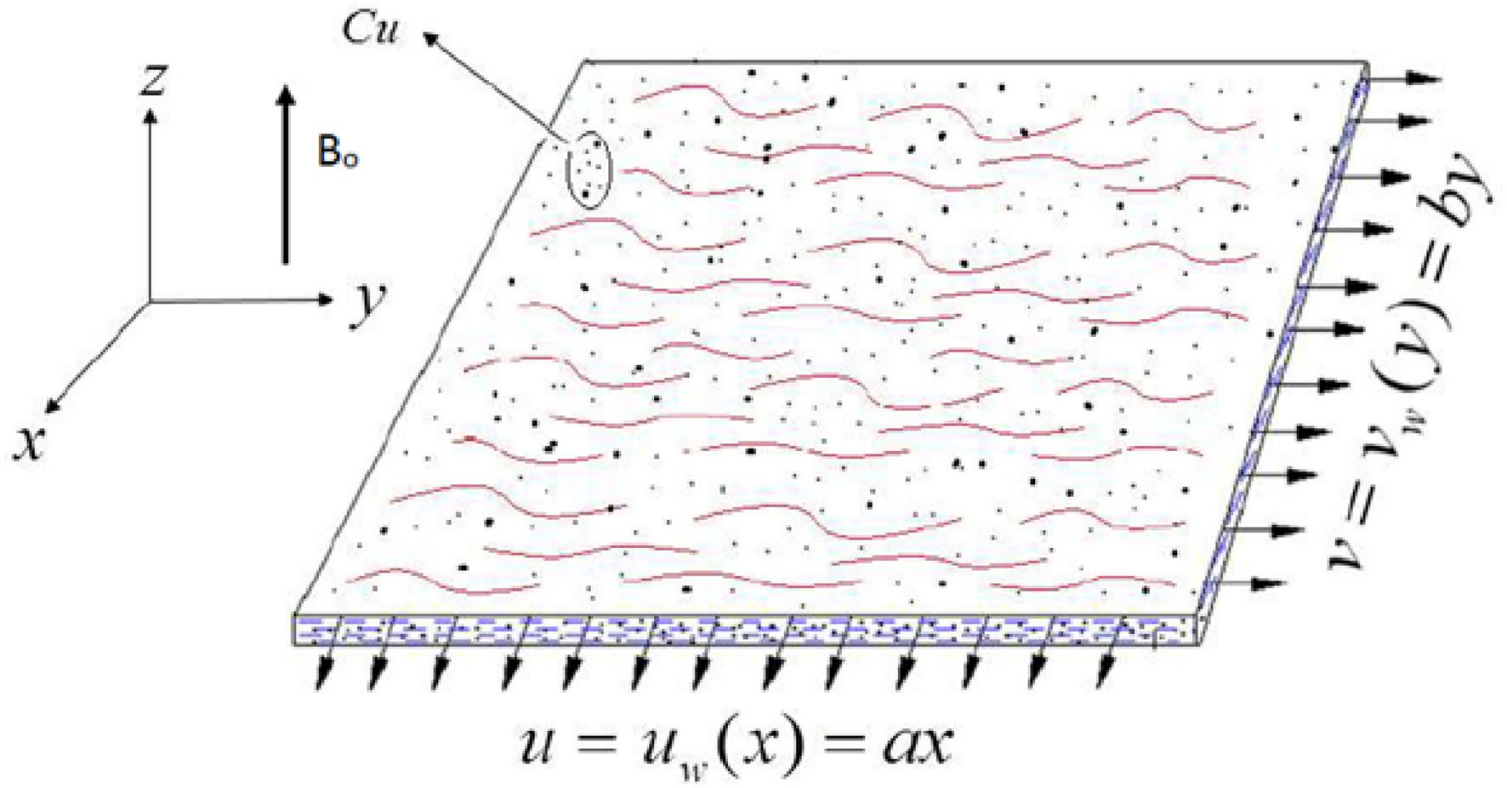
Geometry of the problem.

An incompressible, laminar flow of CNTs is considered over a horizontally held sheet with stretching surface as shown in Fig. 1. A cartesian system (x, y, z) is used here with an even applied magnetic field side by side to z-axis.

After availing the boundary layer approximations, we have obtained the following set of equations for this flow problem:

1 $$\frac{\partial u}{\partial x}+\frac{\partial v}{\partial y}+\frac{\partial w}{\partial z}=0,$$

2 $$\left(u\frac{\partial u}{\partial x}+v\frac{\partial u}{\partial y}+w\frac{\partial u}{\partial z}\right)=\frac{1}{{\rho }_{nf}}\frac{\partial }{\partial y}\left({\mu }_{nf}*\left(T\right)\frac{\partial u}{\partial z}\right)-\frac{\sigma {B}_{0}^{2}}{{\rho }_{nf}}u,$$

3 $$\left(u\frac{\partial v}{\partial x}+v\frac{\partial v}{\partial y}+w\frac{\partial v}{\partial z}\right)=\frac{1}{{\rho }_{nf}}\frac{\partial }{\partial y}\left({\mu }_{nf}*\left(T\right)\frac{\partial v}{\partial z}\right)-\frac{\sigma {B}_{0}^{2}}{{\rho }_{nf}}v,$$

4 $$\left(u\frac{\partial T}{\partial x}+v\frac{\partial T}{\partial y}+w\frac{\partial T}{\partial z}\right)={\alpha }_{nf}\frac{{\partial }^{2}T}{\partial {z}^{2}},$$

The boundary conditions associated with the present problem are

5 $$u={u}_{w}\left(x\right)=ax, v={v}_{w}\left(y\right)=by,w=0, T={T}_{w}, \, at \, z=0, u\to 0, v\to 0, T\to {T}_{\infty }, \, as \, z\to \infty $$

The properties of nanofluids with mathematical modeling are defined^37–39^ as:

6a $${\mu }_{nf}=\frac{{\mu }_{f}{e}^{-\alpha \theta }}{{\left(1-\varphi \right)}^{2.5}},$$

6b $${\alpha }_{nf}=\frac{{k}_{nf}}{{\left(\rho {c}_{p}\right)}_{nf}},{\rho }_{nf}=\left(1-\varphi \right){\rho }_{f}+\varphi {\rho }_{s},$$

6c $${\left(\rho {c}_{p}\right)}_{nf}=\left(1-\varphi \right){\left(\rho {c}_{p}\right)}_{f}+\varphi {\left(\rho {c}_{p}\right)}_{s},$$

6d $${\left(\rho \gamma \right)}_{nf}=\left(1-\varphi \right){\left(\rho \gamma \right)}_{f}+\varphi {\left(\rho \gamma \right)}_{S},$$

6e $${k}_{nf}={k}_{f}\left(\frac{{k}_{s}+2{k}_{f}-2\varphi \left({k}_{f}-{k}_{s}\right)}{{k}_{s}+2{k}_{f}+2\varphi \left({k}_{f}-{k}_{s}\right)}\right)$$

The similarity conversions availed in this analysis are given as:

7 $$\eta =\sqrt{\frac{a}{{\nu }_{f}}}z, u=ax{f}^{^{\prime}}\left(\eta \right), v=by{g}^{^{\prime}}\left(\eta \right),\theta =\frac{T-{T}_{\infty }}{{T}_{w}-{T}_{\infty }},w=\sqrt{\frac{{\nu }_{f}}{a}}\left(af\left(\eta \right)+bg\left(\eta \right)\right)$$

$${\mu }_{nf}={\mu }_{nf}=\frac{{\mu }_{nf}*}{{\mu }_{o}}, M={B}_{0}\sqrt{\frac{a\sigma }{{u}_{w}\rho }}$$

Equations (1)–(5) convey the following reduced form after utilizing the Eqs. (6a–e, 7):

8 $$\frac{{\mu }_{f}\left(\theta \right)}{{\left(1-\varphi \right)}^{2.5}}{f}^{\prime \prime \prime}+\frac{1}{{\left(1-\varphi \right)}^{2.5}}{\mu }_{f}^{^{\prime}}\left(\theta \right){f}^{^{\prime\prime} }+\left[\left(1-\varphi +\varphi \frac{{\rho }_{s}}{{\rho }_{f}}\right)\left\{\left(f+\lambda g\right){f}^{^{\prime\prime} }-{f}^{{^{\prime}}2}\right\}+{M}^{2}{f}^{^{\prime}}\right]=0,$$

9 $$\frac{{\mu }_{f}\left(\theta \right)}{{\left(1-\varphi \right)}^{2.5}}{g}^{\prime \prime \prime}+\frac{1}{{\left(1-\varphi \right)}^{2.5}}{\mu }_{f}^{^{\prime}}\left(\theta \right){g}^{^{\prime\prime} }+\left[\left(1-\varphi +\varphi \frac{{\rho }_{s}}{{\rho }_{f}}\right)\left\{\left(f+\lambda g\right){g}^{^{\prime\prime} }-{g}^{{^{\prime}}2}\right\}+{M}^{2}{g}^{^{\prime}}\right]=0,$$

10 $$\left(\frac{{k}_{nf}}{{k}_{f}}\right){\theta }^{^{\prime\prime} }+\mathit{Pr}\left(1-\varphi +\varphi \frac{{\left(\rho {c}_{p}\right)}_{s}}{{\left(\rho {c}_{p}\right)}_{f}}\right)\left[\left(f{\theta }^{^{\prime}}+\lambda g\right)\right]=0,$$

11a $$f\left(0\right)=0, {f}^{^{\prime}}\left(0\right)=1, {f}^{^{\prime}}\left(\infty \right)=0,$$

11b $$g\left(0\right)=0, {g}^{^{\prime}}\left(0\right)=\lambda , {g}^{^{\prime}}\left(\infty \right)=0$$

11c $$\theta (0)=1, \theta \left(\infty \right)=0,$$

The dimensionless variables appeared here are provided as

$$\mathit{Pr}=\frac{({\mu }_{0}{c}_{p}{)}_{f}}{{k}_{f}}, \lambda =b/a$$

The Reynold model used here for viscosity is^38^:

12 $${\mu }_{f}\left(\theta \right)={e}^{-\left(\alpha \theta \right)}=1-\left(\alpha \theta \right)+O\left({\alpha }^{2}\right),$$

### Skin friction and Nusselt number

13 $${c}_{fx}=\frac{{\mu }_{nf}\left(T\right)}{{\rho }_{f}{{u}_{w}}^{2}}{\left(\frac{\partial u}{\partial z}\right)}_{z=0},{c}_{fy}=\frac{{\mu }_{nf}\left(T\right)}{{\rho }_{f}{{u}_{w}}^{2}}{\left(\frac{\partial v}{\partial z}\right)}_{z=0}N{u}_{x}=\frac{-x{k}_{nf}}{{k}_{f}({T}_{w}-{T}_{\infty })}{\left(\frac{\partial T}{\partial z}\right)}_{z=0}$$

Equation (13) in dimensionless form is given as

14 $$({\mathit{Re}}_{x}{)}^{1/2}{c}_{fx}=\frac{{\mu }_{f}\left(\theta \left(0\right)\right){f}^{^{\prime\prime} }\left(0\right)}{(1-\varphi {)}^{2.5}}, ({\mathit{Re}}_{y}{)}^{1/2}{c}_{fy}=\lambda \left(\frac{y}{x}\right)\frac{{\mu }_{f}\left(\theta \left(0\right)\right){g}^{^{\prime\prime} }\left(0\right)}{(1-\varphi {)}^{2.5}}\text{, }({\mathit{Re}}_{x}{)}^{1/2}N{u}_{x}=-\frac{{k}_{nf}}{{k}_{f}}{\theta }^{^{\prime}}\left(0\right)$$

## Numerical illustration

The shooting technique is employed to numerically expound Eqs. (8)–(10) with specific boundary conditions (11a–11c). The problem was fundamentally a boundary value (BVP) problem and to solve it via shooting technique, we have transformed it into an initial value (IVP) problem. The (RK) Runge Kutta technique with fourth order is employed with some initial guess values for ${f}^{^{\prime\prime} }(0)$, ${\theta }^{^{\prime}}(0)$ and ${g}^{^{\prime\prime} }(0)$ to acquire numerical solutions to the present problem. These initial guess values are better modified via Secant technique. The considered $\Delta \eta =0.01$ is the step size here with a 5th decimal convergence.
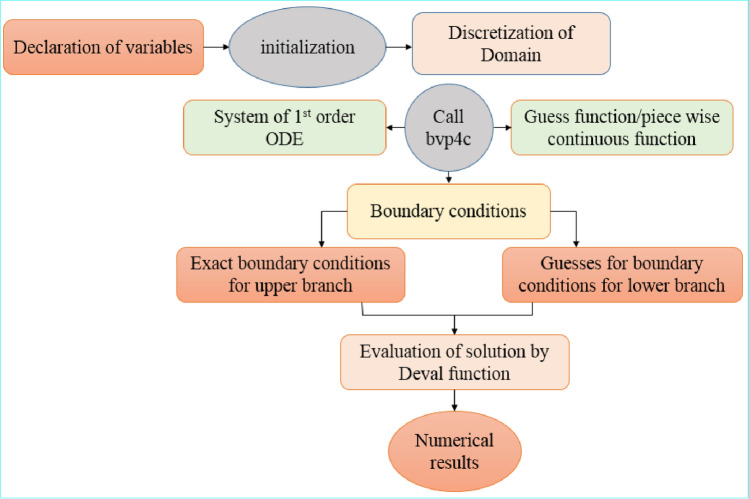


Numerical code to evaluate the solutions is listed below.
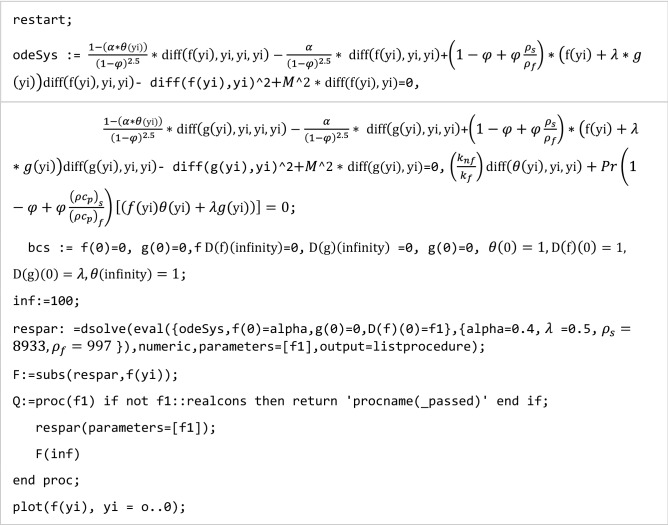


## Results and discussion

**Figure 2:**
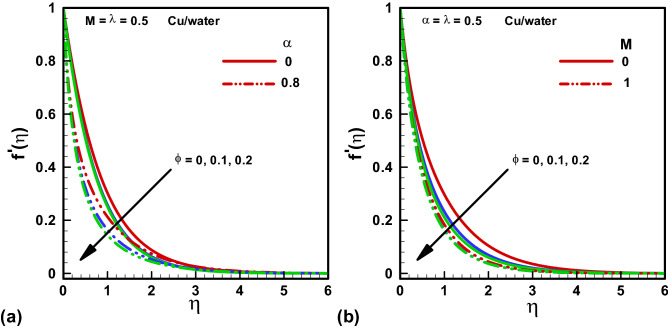
Velocity solutions x-direction for various $\phi$. (**a**) $\alpha =0,$ $\alpha =0.8.$ (**b**) $M=0.$ $M=1.$ Red, Blue, Green Slod lines are for Methanol, Red, Blue, Green dotted lines are for water.

**Figure 3:**
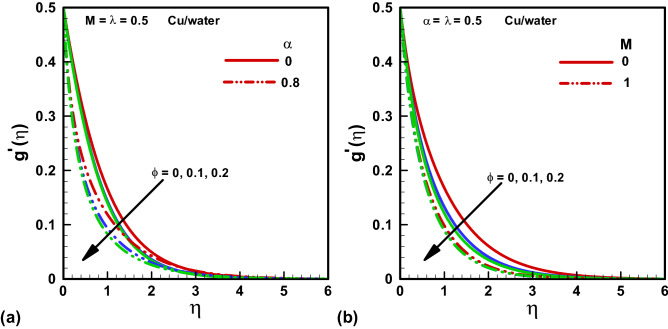
Velocity solutions in y-direction for various $\phi$. (**a**) $\alpha =0,$ $\alpha =0.8.$ (**b**) $M=0.$ $M=1.$ Red, Blue, Green Slod lines are for Methanol, Red, Blue, Green dotted lines are for water.

**Figure 4:**
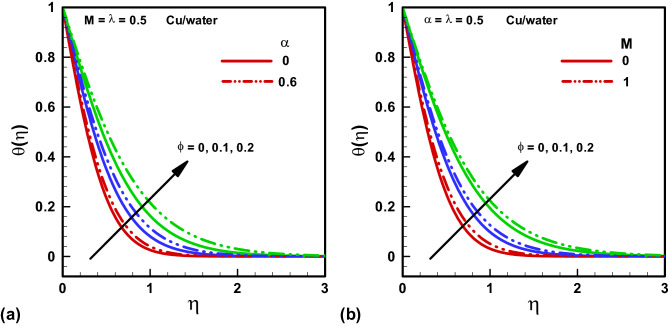
Temperature distribution for various $\phi$ with assisting and opposing flow considering. (**a**) $\alpha =0,$ $\alpha =0.6.$ (**b**) $M=0.$ $M=1.$ Red, Blue, Green Slod lines are for Methanol, Red, Blue, Green dotted lines are for water.

**Figure 5:**
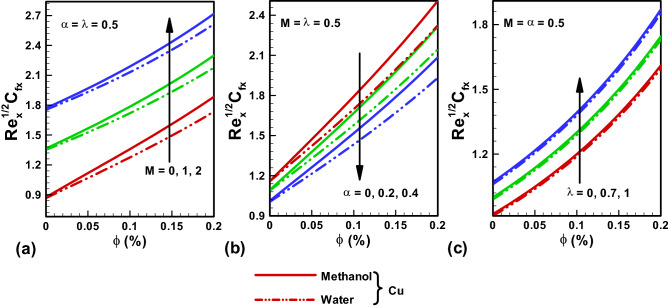
Skin friction results along x-direction (**a**) variable $M$. (**b**) variable $\alpha$. (**c**) variable $\lambda$. Red, Blue, Green Slod lines are for Methanol, Red, Blue, Green dotted lines are for water.

**Figure 6:**
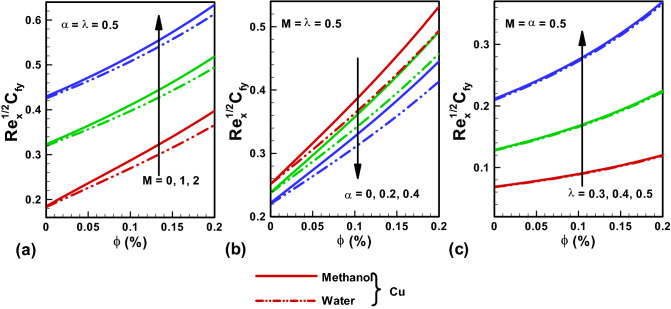
Skin friction results along y-direction (**a**) variable $M$. (**b**) variable $\alpha$. (**c**) variable $\lambda$. Red, Blue, Green Slod lines are for Methanol, Red, Blue, Green dotted lines are for water.

**Figure 7:**
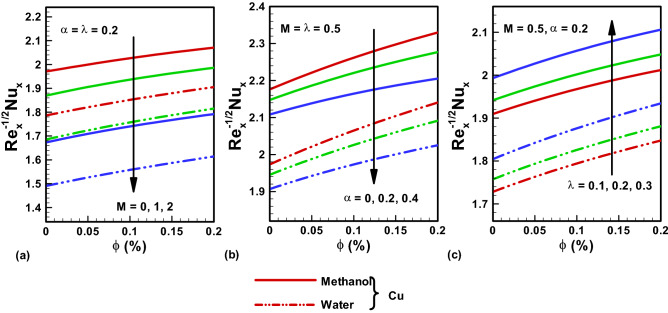
Nu results for (**a**) $M$ (**b**) $\alpha$. (**c**) $\lambda .$ Red, Blue, Green Slod lines are for Methanol, Red, Blue, Green dotted lines are for water.

Figures 2, 3, 4, 5, 6 and 7 depict the graphical conclusions of temperature profile, skin friction, velocity, streamlines and Nusselt number etc. The impact of $M$ and $\alpha$ on the x -direction velocity profile is given by Fig. 2a and b with distinct $\phi =\mathrm{0,0.1,0.2}$ values. The highest velocity profile is observed at $\eta =0$ while it diminishes as $\eta \to \infty$. A declining boundary layer thickness is noted for increasing nanoparticle volume. Further, the flow profile reduces or velocity declines with increasing numerical values of $\alpha .$ The viscosity in our case is varying with changing temperature distribution since a temperature dependent viscosity case is taken here. The incrementing $\alpha$ provides the risen viscous effects those results in enhanced temperature and reduced flow profile. Thus, a declining thickness of the boundary layer is noted. Figure 2b shows that the rising effects of Hartmann number M result in a declining flow profile because the Hartmann number M is the ratio of electromagnetic force to the viscous force. When we increase the Hartmann number electromagnetic force will be dominant to the viscous forces that will decline the velocity profile. Eventually the extent of boundary layer is reduced with incrementing $M.$ Figure 3a and b convey the impact of Hartmann number $M$ and viscosity parameter $\alpha$ on y -direction velocity profile with multiple values of $\phi =\mathrm{0,0.1,0.2}.$ A similar behavior is noted for y -direction velocity profile as that of x -direction.

Figure 4a and b represent the graphical solutions of temperature profile for Hartmann number $M$ and viscosity parameter $\alpha$ with distinct numerical values of $\phi$. An increase in the temperature distribution is observed with distinct incrementing values of the viscosity parameter $\alpha$. Because we are considered temperature dependent viscosity so increasing viscosity parameter $\alpha$ will definitely increase temperature profile. Further, an increase in the extent of thermal boundary layer is observed with increasing Hartmann number $M$. Because when we increase the Hartmann number electromagnetic force will be dominant to the viscous forces that will raise the boundary layer thickness. The incrementing values of temperature viscosity parameter $\alpha$ produce some high fluid resistance and it eventually adds up to a high temperature profile. Thus, an enhanced thermal boundary layer is observed.

Figure 5a–c provide the graphical solutions of skin-friction for multiple flow parameters like $M, \alpha , \lambda .$ Figure 6a–c convey the two distinct considered cases with water and Methanol base fluids. A higher value of skin friction is noted for Methanol in comparison to water. A rise in skin friction is revealed for both cases of base fluid with incrementing $M$. Because when we increase the Hartmann number, the electromagnetic force will be dominant to the viscous forces that will increase the skin friction. Further for both cases of water and Methanol as base fluids, an enhancement in skin friction is noted for enhancing values of the temperature dependent viscosity parameter α. As increasing temperature dependent viscosity parameter α enhance the fluid resistance that will enhance the skin friction coefficient. Further increasing values of α decreases skin friction coefficient both in x and y directions. The high values of ratio of stretching velocities in the y- and x-directions $\lambda$ also result in enhancing skin friction values for both base fluid cases.

Figure 7a–c highlight the graphical illustrations on Nusselt number for multiple parameters. A higher value of Nu is noted for Methanol in comparison to water. Figure 7a depicts a decline in the Nusselt number with rising values of $M$ for both base fluid cases. Thus, Nu declines when a high magnetic field is working as compared to viscous effects. Figure 7b shows that the numerical values of Nu decline for incrementing α. An increase in the value of Nu is observed for incrementing λ as depicted in Fig. 7c.The thermo physical properties of base fluids and Copper are presented in Table 1. Tables 2 and 3 gives the comparison of the present work with existing literature.

**Table 1:** Thermophysical properties of base fluids and copper.

Physical properties	Base fluid		Nanoparticles
	Water	Methanol	Copper
*ρ* (kg/m^3^)	997	792	8933
*c*_p_(J/kg K)	4179	2545	385
*k* (W/m K)	0.613	0.2035	401
$\mathrm{Pr}$	6.2	7.38	–

**Table 2:** Skin friction outcomes comparison for pure fluid with $\alpha =0$ and $\lambda =1$.

$$M$$	$$-{g^{\prime \prime}} (0)$$		
	Present results	Nadeem et al.^35^	Wang^25^
0	1.173721	1.1748	1.173720
10	$3.367240	3.3667	–
100	10.066473	10.0663	–

**Table 3:** Heat transfer rate outcomes comparison for pure fluid with $\alpha =0$ and $\lambda =1, M=0$.

Pr	φ	Wang^25^	Liu et al.^39^	Nadeem et al.^35^	Present work
1	0	0.27741	0.27741	0.27741	0.27741
	0.1	− 0.4496	− 0.4496	− 0.4496	− 0.4496
	0.3	− 0.8547	− 0.8547	− 0.8547	− 0.8547
	0.4	− 1.4602	− 1.4602	− 1.4602	− 1.4602
5	0	1.3532	1.3532	1.3532	1.3532
	0.1	− 1.4212	− 1.4212	− 1.4212	− 1.4212
	0.3	− 1.7001	− 1.7001	− 1.7001	− 1.7001
	0.4	− 1.8021	− 1.8021	− 1.8021	− 1.8021

## Conclusion

A three-dimensional flow analysis is provided for the MHD effects on nanofluids flow over a stretchable surface. This model is applicable in engineering and industrial applications, especially in paper printing and paper production. The numerical solutions are provided for the present research. Some prime outcomes are presented here as:

1. The velocity boundary thickness in x-and y-directions are altered by changes of $M$, $\phi$ and $\alpha$.
2. The extent of thermal boundary layer is also augmented by the rising effect of $M$ and $\alpha$.
3. The coefficient of skin friction in both the x- and y- directions are having relevant changes in magnitude for $M$, $\alpha$ and $\lambda$.
4. The coefficient of skin friction is more for Methanol base fluids in comparison to water base fluid.
5. Nusselt number is rehabilitated with variations in magnetic field, viscosity parameter.
6. Nusselt number is high for Methanol base fluids compared to water base fluid.

## Data Availability

The data sets used and/or analyzed during the current study can be made available from the corresponding author on reasonable request.

## List of symbols

$$(u,v,w)$$: Velocity components, m/s, L/T
$ax$: Linear velocity in x direction, m/s, L/T
B_0_: Magnitude of magnetic field, Tesla = Kg/s^2^A*,* M/AT^2^
$${u}_{w}$$,$${v}_{w}$$: Wall velocities, m/s*,* L/T
${k}_{f}$: Thermal conductivity of the fluid, W/mK, ML/T^3^Θ^1^
${T}_{w}$: Wall temperature, Kelvin*,* $\Theta$
µ_nf_ (*T*): Temperature dependent viscosity of nanofluid, Ns/m^2^, M/LT
*ρ*_*f*_: Density of the base fluid, kg/m^3^, M/L^3^
${k}_{s}$: Thermal conductivity of the nanoparticles, W/mK, ML/T^3^Θ^1^
${\gamma }_{f}$: Thermal expansion coefficient of base fluid, 1/K, I/T
${\gamma }_{s}$: Thermal expansion coefficient of the nanoparticles, 1/K, I/T
${\gamma }_{nf}$: Thermal expansion coefficient of nanofluid, 1/K, I/T
${\rho }_{s}$: Density of the nanoparticles, kg/m^3^, M/L^3^
${k}_{f}$: Thermal conductivity of the base fluid, W/mK, ML/T^3^Θ^1^
$(x,y,z)$: Cartesian system of coordinates, m, L
$by$: Linear velocity in y direction, m/s, L/T
${T}_{\infty }$: Ambient temperature, Kelvin, $\Theta$
$$a,b>0$$: Balancing constants, 1/K, 1/T
$$T$$: Temperature, Kelvin, $$\Theta $$
${\alpha }_{nf}$: Thermal diffusivity of nanofluid, m^2^/K, L ^2^/T
$$\alpha $$: Viscosity parameter (1)
$\lambda$: Ratio of stretching velocities in the y- and x-directions (1)
$$M$$: Hartmann number (1)
θ: Temperature in dimensionless form (1)
Pr: Prandtl number
$\varphi$: Nanoparticle volume fraction
